# Longitudinal associations between non-suicidal self-injury and borderline personality disorder in adolescents: a literature review

**DOI:** 10.1186/s40479-019-0100-9

**Published:** 2019-02-13

**Authors:** Victoria E. Stead, Khrista Boylan, Louis A. Schmidt

**Affiliations:** 1 Department of Psychology, Neuroscience & Behaviour, McMaster University, 1280 King St West, Hamilton, ON L8S 4K1 Canada; 2 Department of Psychiatry and Behavioural Neurosciences, McMaster University, St. Joseph’s Healthcare Hamilton, 100 West 5th, Administration - B3, Hamilton, L8N 3K7 ON Canada; 3 McMaster Children’s Hospital, 1200 Main St. West Hamilton, Hamilton, ON L9N 3Z5 Canada

**Keywords:** Borderline personality disorder, Non-suicidal self-injury, Longitudinal, Adolescents

## Abstract

**Background:**

Borderline personality disorder (BPD) in adolescent samples is similar to BPD in adults concerning clinical characteristics. A notable difference is that adolescents with BPD – and adolescents in general – are more likely than adults to present with acute symptoms such as non-suicidal self-injury (NSSI) and suicidal behaviours. BPD is the only disorder in the Diagnostic and Statistical Manual- 5th Edition that includes a criterion of NSSI. Additionally, NSSI is purported to be a developmental precursor of BPD under the biosocial developmental model. Though much cross-sectional data have illustrated the robust association of adolescent NSSI and BPD, no review to date has summarized the longitudinal associations between these phenomena. The aim of this literature review was to summarize what is known about the longitudinal associations between adolescent NSSI and BPD symptoms. Information on the developmental course of NSSI in relation to BPD would be helpful to clinicians, as the rate of NSSI is high in adolescent populations, and research indicates early, possibly BPD-specific interventions are imperative.

**Methods:**

A literature search was conducted using Embase, MEDLINE, and PsycINFO databases and cited reference searches. Criteria included studies of adolescents (age ≤ 18 at baseline) from either epidemiological or clinical samples, incorporating a longitudinal design, with predictors and outcomes of interest, including both NSSI and BPD diagnosis/symptoms/traits.

**Results:**

Six independent samples were identified that matched our search criteria.

The articles were grouped and reported on separately by population type (epidemiological vs. clinical), and directionality of relations. We identified two epidemiological and four clinical samples. Five samples examined the longitudinal associations of NSSI preceding BPD, three samples measured BPD in adolescence (baseline age ≤ 18), and two of those samples measured BPD at baseline. Both epidemiological studies revealed significant longitudinal associations between NSSI and later BPD/BPD symptoms; however, they differed notably in their methodologies hindering data synthesis across studies. In the clinical studies, findings of the association or predictive relations were not consistent. This is potentially due to differing methodologies, or differences in treatment effectiveness and responsiveness across the samples.

**Conclusions:**

This review highlights the paucity of data that are available examining the longitudinal association between NSSI and BPD within adolescent samples. Thus, it is not possible to reliably comment on how NSSI and BPD are related over time. Future studies will benefit from the measurement of BPD symptoms in very early adolescence, and concurrent measurement of NSSI as well as other forms of suicidal behaviours across adolescence.

## Introduction

Borderline personality disorder (BPD) is a debilitating mental health disorder characterized by patterns of instability and dysfunction across emotional, behavioural, cognitive, and interpersonal domains. BPD in adolescent samples is similar to BPD in adults concerning prevalence, symptom manifestation and course [1–5]. A notable difference, however, is that adolescents with BPD are more likely than adults to present with “acute” BPD symptomatology, such as suicidal ideation, impulsive behaviours, and recurrent non-suicidal self-injury (NSSI), characterized as the deliberate self-inflicted damage and pain to one’s body tissues that is not socially sanctioned and is without suicidal intent [3, 6]. Furthermore, the NSSI/suicidal behaviours criterion is the most frequently met diagnostic criterion in adolescent BPD samples [3, 7–10]. Of particular interest to this study, BPD is the only disorder in the Diagnostic and Statistical Manual- 5th Edition [DSM-5] that includes a criterion of NSSI [11].

### Developmental issues

Research suggests that BPD symptoms peak in late adolescence around 14 to17 years of age [12]. It is also during this time that self-injury, irrespective of intent, is widely prevalent and has become a major health concern with rates of NSSI ranging from 13 to 28% in community samples, and as high as 68 to 80% in inpatient samples [13–15]. We also know from previous cross-sectional studies with adolescents that NSSI and BPD are associated, and this seems to be a robust finding in both clinical [16–20] and community samples [21–23]. More specifically, research has illustrated that BPD symptoms have been shown to be associated with earlier age of onset [10, 23], greater frequency of NSSI [19], and with repeated versus single NSSI episodes [20]. A recent study showed that 95% of previously hospitalized adolescents with BPD reported engaging in self-injury, with 54% engaging in at least 50 episodes [18]. Though there is a strong association between NSSI and BPD, and a high frequency of NSSI in clinical settings, it is recognized that NSSI can occur in individuals who do not have BPD [15, 20, 24].

Though research has confirmed the reliability and validity of a BPD construct in adolescents, it is not regularly made in clinical settings. This is often due to various beliefs held by clinicians about the development of BPD [4, 12, 24–27]. One of the deterrents of making a diagnosis of BPD in adolescents, even in clinical settings where the prevalence of BPD is high, is uncertainty about differential diagnosis. This could be inflated due to high rates of NSSI in these settings [24, 27].

NSSI is purported to be a developmental precursor of BPD pathology under the biosocial developmental model [16, 26, 28–30]. Though previous pivotal studies have mainly focused on the cross-sectional associations of NSSI and BPD in adolescent samples [16–23, 30], longitudinal associations between NSSI and suicide attempts to other mental disorders in adolescents [8, 31, 32], adulthood-spanned BPD samples or retrospective adult data [7, 10], and longitudinal assessment of suicidal behaviours to BPD [24, 33–36]. Thus, NSSI as a developmental precursor of BPD, which requires longitudinal assessment of at least two time points, remains largely underexamined [3, 7, 18, 26, 37, 38].

### Rationale for the study

As illustrated above, there is an abundance of research on the development of BPD, yet there is still a gap in the literature assessing the developmental precursors of BPD, particularly as described in the biosocial developmental model [18, 26, 28, 29, 37]. Cross-sectional research has been valuable in providing us with prevalence rates and associations of NSSI and BPD, but longitudinal studies are necessary to be able to describe the developmental evolution of these phenomena in relation to each other [18, 38]. Thus, this study aimed to synthesize what is known about the longitudinal associations between NSSI, specifically, and BPD in adolescence, which apparently no review has done. Preliminary data have shown that individuals with BPD most likely engage in both NSSI and suicidal behaviours, and these behaviours together might be more useful for identifying BPD [20]. Though, we specifically wanted to focus on only those studies that included clear measurement of NSSI (no suicidal intent), since NSSI may serve a fundamentally different function than self-injury with suicidal intent [30, 39]. Additionally, the diagnostic criteria for depression includes suicidal behaviours (i.e., plans, attempts) not NSSI [11], and we wanted to examine a precursor exclusively related to BPD and free of transdiagnostic overlap. We also recognize that specific personality and temperament constructs (e.g., emotion dysregulation, impulsivity etc.) should precede the development of NSSI behaviours in adolescents; however, again, these personality constructs are transdiagnostic and not unique to BPD criteria [29].

### Clinical implications

Knowledge about the developmental course of NSSI in relation to BPD would be helpful to clinicians, particularly research which can test i) whether NSSI occurs as a developmental precursor as opposed to correlate of BPD, and ii) whether aspects of NSSI (for example, its frequency, methods or associated factors such as substance use) are correlated or interact to predict BPD diagnosis within a defined time interval. This has clinical relevance, as making a valid diagnosis of BPD in clinic attending adolescents who engage in NSSI is critical, since NSSI is highly prevalent in these settings and research indicates early and disorder-specific interventions are most beneficial [3, 27–29, 31, 40].

### Study aims and objectives

The overall aim of this review was to advance the science of BPD in adolescents by examining whether NSSI may be a developmental precursor of adolescent BPD. The first objective was to identify longitudinal studies reporting on the association (predictive or other) between NSSI and BPD symptoms across adolescence in epidemiological and clinical samples. The second objective was to report on how and when NSSI and BPD were measured to comment on whether measurement procedures include consistent, reliable and valid assessment of developmental course. Third, we aimed to summarize what is known about the longitudinal associations of NSSI and BPD/BPD symptoms to be able to comment on whether the data support NSSI as a precursor of BPD. Finally, we attempted to synthesize these findings to derive suggestions for future research.

### Hypothesis

We know from previous work that although intent of self-injurious behaviours is difficult to assess, it is possible to capture, and there are reliable and valid measures that specifically assess NSSI that can be measured in adolescent samples [6, 30, 39]. Additionally, previous research has established the reliability and validity of BPD in adolescents [3, 27, 41]. Given this information and the numerous studies illustrating the strong association between BPD and NSSI in both epidemiological and clincal  studies, and since NSSI is purported to be a precursor of BPD, we hypothesized that we would find longitudinal studies that appropriately measure NSSI and BPD during the developmental period of adolescence that would provide support for NSSI as a precursor of BPD.

## Method

### Selection criteria

Participants, Interventions, Comparisons, Outcomes (PICO) [42]. A Study Design approach was used to generate the research question. Our inclusion criteria were studies of adolescents (sample age ≤ 18 at baseline) using a longitudinal design. Treatment studies (effect modification) and case studies were excluded. The predictors and outcomes of interest included any measure of NSSI, BPD diagnosis, and BPD symptoms/traits. Only studies that examined NSSI behaviours, and not the proposed NSSI disorder, were included in this review. Studies were eliminated during the screening process if their self-injury variables included any acts with reported suicidal intent, combined behaviours with and without suicidal intent, and unclear suicidal intent (i.e., no confirmation). This was to ensure the examination of NSSI in isolation of suicide attempts. Due to our strict inclusion criteria, the Avon Longitudinal Study of Parents and Children (ALSPAC) cohort, (see Lereya et al., 2013) [43] was not included as the self-injury variable included acts with and without suicidal intent.

### Information sources and search

A search of electronic databases was performed including PsycInfo (1804-June 29, 2018), MEDLINE(R) (1946-June 29, 2018), and Embase (1974-June 29, 2018). Non-English publications were excluded. We specifically wanted to evaluate the literature on adolescent samples. Studies that did not measure either NSSI or BPD at least once prior to age 18 were excluded.

We first searched NSSI and related key terms that included: “injury,” “non-suicidal,” “non-suicidal self-injury,” “NSSI,” “non-suicidal self-harm,” “self-destructive behaviour,” “self-inflicted wounds,” “self-injurious behaviour,” “self-mutilation,” “self-harm.” Next, we added key terms for borderline personality disorder by including: “borderline personality disorder,” “borderline states,” “borderline personality symptoms,” “borderline personality features,” “BPD,” and “borderline*.” Lastly, we only wanted to examine adolescent samples, so we included the following key terms into our search: “adolescence,” “adolescents,” “adolescent development,” “adolescent psychopathology,” “teens,” and “youth.”

The screening process was conducted independently by the lead author (V.E.S.) based on the study inclusion criteria outlined above. The titles and abstracts were screened to eliminate non-relevant and duplicate studies. The full texts of the remaining studies were examined against inclusion criteria. If there was any uncertainty about the inclusion of a study, the other two authors (K.B. and L.A.S.) were consulted for their opinions.

## Results

**Fig. 1:**
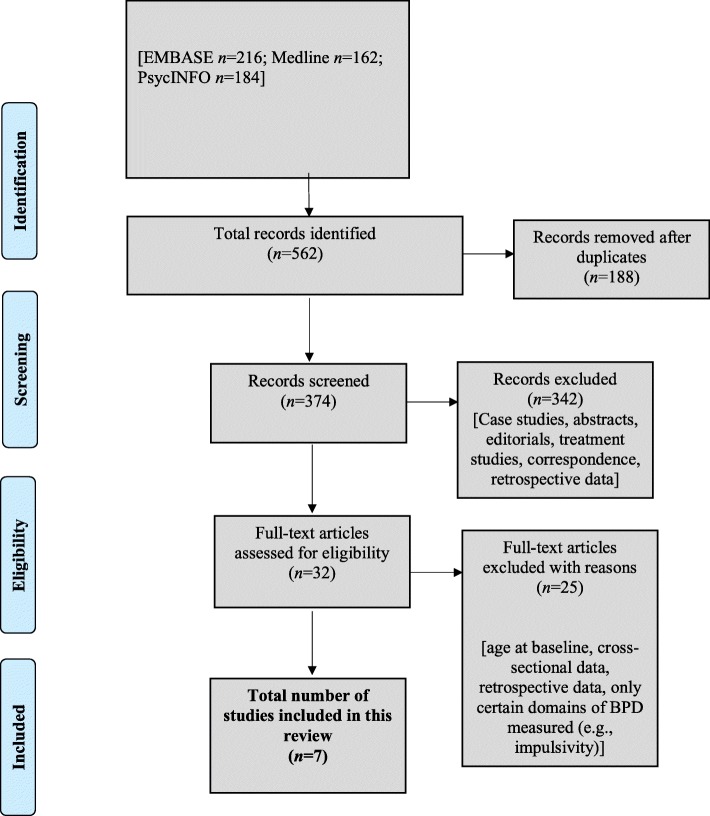
Flowchart of study selection process

Overall, our search produced 562 citations. After removing duplicates, and screening titles and abstracts, 32 complete articles were reviewed, and a total of seven articles met our inclusion criteria (see Fig. 1). Of the seven articles, there were six independent samples (two epidemiological and four clinical). The articles were grouped and reported on separately by population type (epidemiological vs. clinical). To further synthesize findings, we analyzed studies by grouping them on the temporal directionality of the predictor and outcome of interest (e.g., NSSI preceding BPD/BPD symptoms vs. BPD/BPD symptoms preceding NSSI) if NSSI and BPD were not measured at each time point.

### Epidemiological studies

#### NSSI predicting BPD (see Table 1)

Nakar et al. (2016) examined developmental trajectories of NSSI, suicidal behaviours, and substance misuse in relation to BPD traits later in adolescence. The authors examined adolescents from 26 German schools, using a school randomization sampling model (*N* = 513; 62% females; mean age at baseline = 14.5) [44]. NSSI was assessed using a modified version of the Deliberate Self-Harm Inventory (DSHI), which measured the prevalence and frequency of NSSI. Participants were asked about lifetime prevalence of NSSI at baseline and past year prevalence in the second and third waves [45]. An ordinal coding scheme was applied to each time point: never engaging in NSSI, occasional NSSI classified as less than five times, and repetitive NSSI classified as five or more occurrences. Participants were then grouped into low, moderate and high-risk groups based on their NSSI endorsement profile across all time points. BPD traits were measured using the 15-item BPD screener and the interview from the Structured Clinical Interview for DSM-IV Axis II Personality Disorders (SCID-II-PQ) at the final wave, two years from the baseline assessment [44, 46].

**Table 1:** Epidemiological studies

Directionality of associations	Study’s author(s), year	Country	*N* at follow-up (retention rate), % females and mean (*M*) age at baseline	Follow-up period	Measure (s)	BPD measured at baseline; age of BPD assessment	Findings
NSSI predicting BPD	Nakar et al., 2016	Germany; 26 schools – The Saving and Empowering Young Lives in Europe study	*N* = 513 (36%); 62% females; *M*age = 14.5 years	2 years (3 assessments)	DSH Inventory SCID-II (15-item BPD screener and interview)	No; *M*age = 14.5 years at baseline + 2 years	Statistically significant differences on number of BPD criteria scores between low, moderate and high risk NSSI groups (*F* (2,510) = 1229.584, *p* < 0.001, η^2^ = 0.828), with a mean of 7.33 BPD criteria for the high-risk group NSSI (overall mean number of BPD criteria = 3.62).
NSSI and suicidal ideation predicting BPD	Scott et al., 2015	USA	*N* = 1950 (80%), includes 4 cohorts; all females; age at baseline = 5–8 years	6–9 years 1st SI measure at age 10 1st NSSI measure at age 13 both measures assessed until ages 16–19	CSI-4 ASI-4 ASRI-4 SCID-I IPDE-BOR (screening questionnaire)	No; Ages 16–19	Those who reported SI-only and SI + NSSI both had significantly more BPD symptoms than those with no SI or NSSI. And the SI + NSSI group reported significantly more BPD symptoms than the SI-only group. Observed range of BPD scores 0–8; No SI or NSSI *M* = 1.2, *SD* = 1.64; SI-only *M* = 2.22, *SD* = 1.99; SI + NSSI *M* = 2.84, *SD* = 2.05, (*F* (2,1947)= 70.30, *p* < .001).

*Note. BPD* borderline personality disorder, *NSSI* non-suicidal self-injury, *SI* suicidal ideation, *DSH* The Deliberate Self-Harm Inventory, *SCID-II-PQ* The Structured Clinical Interview for DSM-IV Axis II Personality Disorders, *CSI-4* Child Symptom Inventory 4th-edition, *ASI-4* Adolescent Symptom Inventory 4th-edition, *ASRI-4* Adult Self-Report Inventory 4th-edition, *SCID-I* The Structured Clinical Interview for DSM-IV Axis I, *IPDE-BOR* The International Personality Disorder Examination-Borderline

##### Results

The high-risk NSSI group (*n* = 81) had a greater number of BPD traits (7.33 symptoms) compared to the low and medium-risk groups. Additionally, the high-risk NSSI group decreased in NSSI behaviours over time. The researchers also examined suicidal behaviours and substance misuse. They reported that there was a high degree of overlap between participants in the high-risk groups for all three phenomena, and this overlap was associated with elevated levels of BPD traits. The same analysis examining the high-risk NSSI group (*n* = 50) in relation to later BPD traits as measured by the SCID-II interview (as opposed to the SCID-II screening questionnaire) was not significant [44].

#### NSSI and suicidal ideation predicting BPD (Table 1)

Scott et al. (2015) examined NSSI and suicidal ideation in predicting adolescent BPD in a high-risk urban community sample of adolescent females in Pittsburgh, USA (the Pittsburgh Girls Study, *N* = 1950) [47]. The focus of their study was to examine histories of suicidal ideation-only and combined suicidal ideation+NSSI during early to late adolescence as prospective predictors of suicide attempts. Participants were between five and eight years old at the intake assessment, and this study included an 11-year, annual follow-up design to age 19.

Suicidal ideation was assessed using the Child Symptom Inventory (age 10), the Adolescent Symptom Inventory (age 12), and the Adult Self-Report Inventory (age 19). Suicidal ideation was considered present at each wave of the study (age 10–19) if a participant endorsed “sometimes” in the past year to suicidal ideation [48–50]. Of the sample, 40% reported experiencing suicidal ideation at some point between ages ten and 19. NSSI was captured using items from the Structured Clinical Interview for DSM Disorders, Research Version, Non-patient Edition (SCID-I) first at age 13 and again at any point up to and including age 19 [51]. NSSI was coded as being present if it was endorsed at any point between age 13 and 19. Only 1% of the sample endorsed NSSI-only, so the authors excluded this group from their primary analyses. Approximately 9% of the included sample endorsed NSSI. Participants were then grouped based on what behaviours they endorsed as follows: no behaviours (60%), suicidal ideation-only (31%), and suicidal ideation+NSSI (9%) during this age ten to 19 window.

BPD was assessed when participants were ages 16 to 19, using the The International Personality Disorder Examination-Borderine (IPDE-BOR) screening questionnaire [52]. Importantly, items related to NSSI and suicidal ideation were dropped from the total BPD calculation, an item measuring identity confusion was added, and then all items were summed to create a dimensional score of BPD symptoms.

##### Results

There was an association between the no behaviours, suicidal ideation-only, and combined suicidal ideation+NSSI groups in predicting BPD symptom scores. Results indicated that girls who endorsed both suicidal ideation+NSSI had significantly more BPD symptoms than those with suicidal ideation-only and both groups had significantly more than controls. Thus, the presence of both suicidal ideation and NSSI at any time in adolescence was associated with greater BPD severity at age 16 to 19 [47].

### Clinical studies

#### NSSI predicting BPD (see Table 2)

Four studies examined NSSI in predicting later BPD. These studies included: Groschwitz et al. (2015), Homan et al. (2017), and Koenig et al. (2017, 2018) [26, 53–55]. Of note, Koenig et al. (2017, 2018) reported on the same sample, and the authors were primarily interested in examining pain sensitivity in adolescents with NSSI and resting-state cardiac function and BPD in adolescents who engage in NSSI, respectively [54, 55]. Groschwitz et al. (2015) screened psychiatric clinic attendees’ files for terms related to NSSI, and those with at least one episode of NSSI in their medical record were contacted [53]. Participants from the Homan et al. (2017) study were recruited from an inpatient psychiatric unit for self-injury and suicide-related behaviours. The patients’ medical records were examined from discharge to five-year follow-up for any diagnosis of BPD or BPD traits [26]. Koenig et al. (2017, 2018) recruited participants from a specialized outpatient clinic for risk-taking and NSSI behaviours. These studies varied in the number of participants, ranging from 17 to 116 adolescents at follow-up. Participants were 14 to 16 years of age at baseline. The majority of the samples were females (71–100%). The follow-up periods of the studies ranged from one to eight years [26, 53–55].

**Table 2:** Clinical studies

Directionality of associations	Study’s author(s), year	Country	*N* at follow-up (retention rate), % females and mean (*M)* age at baseline	Follow-up period	Measure(s)	BPD measured at baseline; age of BPD assessment	Findings
NSSI predicting BPD	Groschwitz et al., 2015	Germany (terms related to NSSI reported in clinical record were criteria for being recruited for study)	*N* = 52 (73%); 94.2% females; age of onset of NSSI was 13.9 years	8 years *M*age at follow-up = 21.5 years	Clinically verified record of NSSI SITBI-G SCID-II	No; *M*age = 21.5 years	No significant association between NSSI group status (prevailing vs. ceased) and later BPD (χ^2^= 0.265, *p* = 0.78). However, earlier age of onset of NSSI (*Z* = 2.699, *p* = 0.007, *d* = 0.82) and longer duration of NSSI (*Z* = 2.93, *p* = 0.003, *d* = 0.94) during adolescence predictive of adult BPD.
	Homan et al., 2017	USA (recruited for NSSI and suicide behaviours)	*N* = 116 (88%); 71% females; *M*age = 16	5 years	SHBQ Clinic records assessed for BPD diagnosis and traits	No; *M*age = 23.6 years	NSSI did not predict BPD 5 years later in univariate model or in multivariate model that included SA, SI, and ST (OR = 1.42, 95%CI: 0.83–2.44, *p* = .20). The overall multivariate model (NSSI, SI, SA, ST) was significant (χ^2^ = 9.52, *p* = .05).
	Koenig et al., 2017 (same sample as Koenig et al., 2018)	Germany (recruited for risk-taking and NSSI)	*N* = 18 (60%); all females; *M*age = 15.3	1 year	SITBI-G SCID-II- German version	Yes; *M*age = 15.36 years	Participants were recruited for engaging in NSSI. There was a significant decrease in NSSI frequency from baseline to 1-year follow-up (χ^2^_(18)_= 15.95, *p* < 0.001). There were no significant changes in number of BPD criteria met (χ^2^_(18)_= − 3.12, *p* = 0.078), or the number of individuals meeting diagnostic criteria for BPD (χ^2^_(18) =_ 2.00, *p* = 0.289) over time. Thus, NSSI predicts stability of BPD diagnosis and symptoms in this sample.
	Koenig et al., 2018 (same sample as Koenig et al., 2017)	Germany (recruited for risk-taking and NSSI)	*N* = 17 (60%); all females; *M*age = 15.3	1 year	SITBI-G SCID-II- German version	Yes; *M*age = 15.3 years	There were no significant differences in the specific BPD symptoms that were endorsed at baseline and 1-year follow-up (p’s > .1). Thus, NSSI predicts stability of BPD criteria symptoms within this sample.

*Note. BPD* borderline personality disorder, *NSSI* non-suicidal self-injury, *SA* suicide attempts, *SI* suicidal ideation, *ST* suicide threats, *SITBI-G* Self-Injurious Thoughts and Behaviors Interview-German version, *SCID-II* the Structured Clinical Interview for DSM-IV Axis II Personality Disorders, *SHBQ* The Self-Harm Behaviour Questionnaire

*Note.* *BPD* borderline personality disorder, *NSSI* non-suicidal self-injury, *SA* suicide attempts, *SI* suicidal ideation, *ST* suicide threats, *SITBI-G* Self-Injurious Thoughts and Behaviors Interview-German version, *SCID-II* the Structured Clinical Interview for DSM-IV Axis II Personality Disorders, *SHBQ* The Self-Harm Behaviour Questionnaire

The semi-structured Self-Injurious Thoughts and Behaviors Interview- German version (SITBI-G) was administered in three of the studies to assess NSSI and suicide attempts both present and lifetime [53–56]. Participants in the Groschwitz et al. (2015) study were split into two groups, those who reported at least one episode of NSSI within the last year (self-injury prevailing; *n* = 24), and those who had not engaged in NSSI for at least one year prior to the interview (self-injury ceased; *n* = 28). Koenig et al. (2017, 2018) included participants into their study who endorsed at least five incidents of NSSI during the past 12-months (consistent with DSM-5 section 3 diagnostic criteria for NSSI). Lastly, Homan et al. (2017) assessed NSSI via the Self-Harm Behavior Questionnaire (SHBQ), which is a 32-item self-report measure used to assess the frequency and severity of NSSI [57, 58]. This measure is divided into four sections assessing NSSI, suicide attempts, suicidal ideation and suicide threats [26].

The SCID-II was used to assess for BPD diagnosis in three of the studies [53–55]. However, Homan et al. (2017) assessed BPD through medical record documentation of BPD and BPD traits. BPD was considered a valid diagnosis once participants were 18 years old. Both those patients who were diagnosed with definite and probable BPD were assigned to the BPD group (*n* = 25) [26].

##### Results

Overall, two studies reported no significant associations between NSSI and later BPD. Groschwitz et al. (2015) found no associations between current NSSI group status and BPD diagnoses (χ^2^= 0.265, *p* = 0.78). Half of those with BPD reported NSSI within the past year of the eight-year follow-up assessment, and half did not. However, earlier age of onset in adolescence (*Z* = 2.699, *p* = 0.007, *d* = 0.82) and longer duration of engagement of NSSI in adolescence (*Z* = 2.93, *p* = 0.003, *d* = 0.94) were predictive of adult BPD within this sample [53]. Similarly, Homan et al. (2017) did not find an association between adolescent NSSI and later adult BPD as a predictor in the univariate or multivariate analyses *(OR* = 1.42, 95%CI: 0.83–2.44, *p* = .20). Conversely, Koenig et al. (2017, 2018) found that each BPD criteria symptom assessed independently (*p*’s > .1), number of BPD symptoms met (χ^2^_(18)_ = − 3.12, *p* = 0.078), and number of individuals meeting diagnostic criteria for BPD (χ^2^_(18) =_ 2.00, *p* = 0.289) to be stable over the year in their sample of adolescents with NSSI behaviours. Though, there was a significant decrease in NSSI from baseline to one-year follow-up (χ^2^_(18)_= 15.95, *p* < 0.001) in these adolscents [26, 53–55].

#### BPD predicting NSSI (see Table 3)

Yen et al. (2016) examined adolescent BPD predicting NSSI over a six-month period. Their study aimed to assess prospective predictors of NSSI in adolescents who were admitted to an inpatient psychiatric unit in the United States. Participants were recruited for presenting with increased suicide risk (i.e., recent suicide attempt, NSSI with suicidal ideation, or suicidal ideation), and 78 individuals completed data for the full study (68% females; mean age at baseline = 15.1 years) [59].

**Table 3:** Clinical studies

Directionality of associations	Study’s author(s), year	Country	*N* at follow-up (retention rate), % females and mean (*M*) age at baseline	Follow-up period	Measure (s)	BPD measured at baseline; age of BPD assessment	Findings
BPD predicting NSSI	Yen et. al, 2016	USA (inpatient- recruited for suicide risk)	*N* = 78 (77%); 68% females; *M*age = 15.1	6-months	CI-BPD FASM	Yes; *M*age = 15.1 years	BPD diagnosis (with NSSI/suicide criterion removed) did not predict NSSI at 6-month follow-up (χ^2^ = .19, *p* < .05). These results remained with all 9 BPD criteria included.

*Note. BPD* borderline personality disorder, *NSSI* non-suicidal self-injury, *CI-BPD* Childhood Interview for DSM-IV Borderline Personality Disorder, *FASM* The Functional Assessment of Self-Mutilation

The Childhood Interview for DSM-IV Borderline Personality Disorder (CI-BPD) was used to assess BPD at baseline and six-month follow-up, and was administered to both parents and adolescents separately. The NSSI/suicide criterion was removed from the total score. The Functional Assessment of Self-Mutilation (FASM) was used to assess methods, frequency and current use of NSSI within the past year [60]. The authors adapted this to be able to capture their six-month follow-up. Behavioural functions of NSSI were also assessed (e.g., automatic reinforcement vs. social reinforcement). Persistence of NSSI was defined as the endorsement of NSSI at both baseline and six-month follow-up using data from all measures (i.e., FASM, CI-BPD, phone check-ins, and clinical reports).

##### Results

In total, 28% of the sample met criteria for BPD. 54% of those with BPD had persistent NSSI over the six-month period, and 51% of the total sample endorsed persistent NSSI. BPD diagnosis status (with NSSI/suicide criterion removed) did not predict persistence of NSSI at the six-month follow-up (χ^2^ = .19, *p* < .05). These results remained when all nine BPD criteria symptoms (including self-injury/suicide) were included in their BPD score [59].

## Discussion

The first objective of this review was to determine whether studies have measured the longitudinal associations of NSSI and BPD/BPD symptoms in adolescent samples. We identified seven studies (six distinct samples) with longitudinal data with at least one measure of NSSI and BPD in adolescence. The second objective was to report on how and when NSSI and BPD were measured. We hypothesized that at least some of these longitudinal studies would measure BPD and NSSI repeatedly across the developmental period of adolescence, allowing us to test the hypothesis that NSSI may be a precursor of BPD. However, NSSI and BPD were not consistently measured concurrently within studies, limiting confidence in the overall findings.

Five samples examined the longitudinal association of NSSI preceding BPD [26, 44, 47, 53–55], three samples measured BPD in adolescence (baseline age ≤ 18) [44, 54, 55, 59], and two of those samples measured BPD at baseline [54, 55, 59]. Consequently, the most notable gap from this literature is the absence of measurement of adolescent BPD symptoms when NSSI is measured for the first time. Without concurrent baseline measurement of NSSI and BPD in adolescence, it is not clear whether NSSI strongly predicts BPD symptoms, or is simply a correlate [38, 44, 47]. Considering studies have validated the adolescent BPD diagnosis via the CI-BPD with children as young as 11 years old, future work can and should strive to capture early emerging phases of BPD by accurately measuring BPD symptomatology as early as possible in adolescence [12, 27, 61].

Method of BPD measurement differed across studies (e.g., continuous variables vs. dichotomous variables, interviews vs. screeners vs. medical charts), and sometimes yielded different results. It is possible that examining BPD continuously is more representative of the adolescent presentation, as a dynamic spectrum may capture emerging pathology and prodromal individuals who are also impaired and engage in NSSI [35]. Additionally, when BPD screening tools reveal significant results compared to their interview counterparts, we need to be careful with our interpretations, as screening tools for BPD might not be specific enough at detecting true negatives and thus confounding the results [44].

NSSI was measured in different ways, and sometimes split into what seemed like arbitrary groups (e.g., occasional NSSI being less than five occurences vs. repetitive NSSI being five or more occurrences; those who reported at least one episode of NSSI within the last year vs. no NSSI) [44, 53]. In epidemiological studies, this type of coding might be useful and valid [62], but likely under-represents the range of NSSI frequency and does not capture patterns of NSSI in clinical samples [18]. A recent study has shown that by examining the frequency and time between NSSI acts, specific patterns of NSSI might be more suggestive of BPD in youth (15–25 years) [63]. Additionally, another study found that earlier age of onset and longer duration of NSSI predicted later BPD [53]. Taken together, this emphasizes the importance of precisely measuring frequency, duration, and number of NSSI methods, as it appears specific NSSI patterns may demonstrate clinically essential differences for predicting BPD in adolescents [53, 63].

The studies varied considerably in time to follow-up. Clinical studies are often arduous to conduct and have high attrition rates. However, relatively short follow-up periods (six-months- one year) might not be enough time to elucidate specific behavioural patterns of change associated with adolescent BPD. Additionally, the clinical studies included participants receiving varying types of treatment, and it was unclear, for some of the studies, if and how these different treatments were controlled for in overall analyses [26, 53, 59]. It is possible that the results reflect the effect of treatment on BPD symptoms (and subsequent NSSI behaviours in adolescents with BPD). These points are especially important to consider, since BPD symptomatology is shown to be highly reactive to environmental cues, as rates of BPD symptom fluctuations are seen to be associated with situational factors for these adolescents [4, 28, 29, 64]. In summary, across studies, there were inconsistencies around the timing of measurement (i.e., age and developmental period at which the phenomena were measured), the directionality of the relations, variables not measured in conjunction with each other over time, and how phenomena were operationalized or measured.

Our third objective was to summarize what is known about the longitudinal associations of NSSI and BPD/BPD symptoms to be able to comment on whether the data support NSSI as a precursor of BPD. From the epidemiological studies, it would be inaccurate to comment on developmental patterns, due to the vastly differing methodologies across studies, and because BPD was not measured at baseline. Therefore, it is not clear whether BPD preceded or co-occurred with NSSI. From the clinical studies, findings about the association or predictive relation were not consistent. This again may be due to the differing methodologies, or because of differences in treatment effectiveness and responsiveness across the samples. Overall, this review highlights the many gaps in the literature assessing the longitudinal association between NSSI and BPD. Due to the small number of studies and inconsistent study methodologies and findings, it is unclear whether NSSI is a precursor of BPD.

### Future directions

To best characterize the relation between NSSI and BPD symptoms in adolescents, multi-year prospective studies with at least three time points are needed that measure NSSI and BPD symptoms at each wave of the study. Additionally, including and examining both epidemiological and clinical samples is important, as NSSI is highly prevalent in both of these samples, and intergroup differences on developmental precursors are plausible. Precise measurement of methods, frequency, and functions of NSSI, and the other behaviours of the BPD NSSI/suicide criterion (i.e., suicidal behaviours, gestures, and threats), in conjunction with suicidal ideation, should improve prediction. These behaviours are clinically related, and their co-occurrence probably best predicts BPD. In line with the work of Nakar et al. (2016), measurement of substance use, and potentially other more socially acceptable emotion dysregulation behaviours may also provide fundamental information about symptom trajectory profiles of BPD in adolescence, and therefore should be also included [3, 44]. Studying all of these emotion dysregulation behaviours together could provide useful information about early detection of BPD.

The age range of the adolescent sample needs to be considered carefully when comparing studies measuring NSSI and BPD together. There is a peak in symptoms of both NSSI and BPD in adolescence, and these symptoms may be more strongly correlated during this time [12, 13]. Some studies examined NSSI and BPD in youth 13 to 21 or 15 to 25 years of age, and were not included in our review, due to sample age crossing into young adulthood [7, 63]. Developmental stage needs to be considered when examining the developmental course of these phenomena, especially since BPD is conceptualized as a developmental disorder [28]. There are important developmental (e.g., physiological, neurobiological, environmental, etc.) changes that occur during adolescence that warrant BPD and other clinical features to be examined in isolation within this developmental stage, as these changes in biological and environmental systems probably have important implications on behavioural symptom presentations [65–67].

## Conclusion

To optimally investigate the developmental precursors of BPD, researchers should employ prospective research designs that incorporate many aspects of the biosocial model of BPD within a developmental framework [28, 29]. This review highlights the need for concurrent measurement of BPD symptoms and common indicators of emotion dysregulation, such as NSSI, suicidal behaviours and substance use across the developmental window of adolescence. This measurement work is critically important to delineate the developmental precursors associated with a coherent and persistent BPD syndrome in adolescents [63]. This cited research is the foundation for the addition of other tests of the biosocial developmental model of BPD, namely biological (e.g., heart rate variability, temperament/personality etc.), and environmental (e.g., current life situations, treatment, etc.) factors.

## Abbreviations

ASI-4: Adolescent Symptom Inventory 4th-edition
ASRI-4: Adult Self-Report Inventory 4th-edition
CI-BPD: Childhood Interview for DSM-IV Borderline Personality Disorder
CSI-4: Child Symptom Inventory 4th-edition
DSH: The Deliberate Self-Harm Inventory
FASM: The Functional Assessment of Self-Mutilation
IPDE-BOR: The International Personality Disorder Examination
NSSI: Non-suicidal self-injury
SA: Suicide attempts
SCID-I: The Structured Clinical Interview for DSM-IV Axis I
SCID-II: The Structured Clinical Interview for DSM-IV Axis II Personality Disorders
SHBQ: The Self-Harm Behaviour Questionnaire
SI: Suicidal ideation
SITBI-G: Self-Injurious Thoughts and Behaviors Interview- German version
ST: Suicide threats
